# Supplementary material for: Structure of the two-component S-layer of the archaeon Sulfolobus acidocaldarius
Source: eLife. 2024 Jan 22;13:e84617. doi: 10.7554/eLife.84617 (PMC10903991; doi:10.7554/eLife.84617)
Supplement: Supplementary file 1. — (a) Statistics of data collection, 3D reconstruction, and validation. (b) Mapping of glycan residues from the structure file to residues of the GLYCAM force field or the newly charge-derived SG0 and SG4 residues, representing the 1-substituted and 1,4-substituted SMA. (c) RESP charges derived for residue SG0 on the HF/6-31G*//HF/6-31G* level of theory (see Methods for details). (d) RESP charges derived for residue SG4 on the HF/6-31G*//HF/6-31G* level of theory (see Methods for details). [file elife-84617-supp1.docx]

|  | **Dataset pH 4** | **Dataset pH 7** | **Dataset pH 10** | **CryoET Dataset 1** | **CryoET Dataset 2** |
| --- | --- | --- | --- | --- | --- |
| **Data collection** |  |  |  |  |  |
| Electron microscope | FEI Talos Arctica | FEI Talos Arctica | FEI Talos Arctica | FEI Talos Arctica | Thermo Fisher Titan Krios |
| electron detector | Gatan K2 Summit | Gatan K2 Summit | Gatan K2 Summit | Gatan K2 Summit | Thermo Fisher Falcon 4i |
| Voltage (kV) | 200 | 200 | 200 | 200 | 300 |
| Defocus range (μm) | -2.4 to -1.2 | -2.2 to -1 | -2.4 to -0.8 | -4 to -6 | -4 to -6 |
| Pixel size (Å^2^) | 1.05 (0.525) | 1.05 (0.525) | 1.05 (0.525) | 2.21 (1.105) | 1.9 |
| Total electron dose (e^-^/ Å^2^) | 59.12 | 58.465 | 61 | 83.64 | 83 |
| Exposure time (s) | 11 | 11 | 12 |  |  |
| Number of fractions | 44 | 44 | 60 | 2 | 6 |
| Total movies/tomograms | 3,687 | 5,328 | 5,046 | 26 | 58 |
| **3D reconstruction** |  |  |  |  |  |
| Initial particles | 1,419,989 | 1,574,035 | 374,916 | 22,950 | |
| Final particles | 66,040 | 90,965 | 29,819 | 2,771 | |
| Resolution (masked FSC=0.143; Å) | 3.1 | 3.9 | 3.2 | 11.2 | |
| B factor | -58.49 | -116.58 | -69.44 |  | |
| EMDB accession # | EMD-14635 | EMD-15531 | EMD-15530 | EMD-18127 | |
| **Model refinement** |  |  |  |  |  |
| PDB ID | 7ZCX | 8AN3 | 8AN2 | 8QP0 and 8QOX | |
| Model resolution (FSC=0.50/0.143; Å) | 3.1/2.7 | 3.7/3.3 | 3.1/2.8 |  |  |
| Model refinement resolution (Å) | 3 | 3.8 | 3.2 |  |  |
| Non-hydrogen atoms (overall/protein/glycan) | 8895/7826/1069 | 8721/7826/895 | 8657/7826/831 |  |  |
| **RMS deviations** |  |  |  |  |  |
| Bond length (Å) | 0.009 | 0.008 | 0.01 |  |  |
| Bond angle (°) | 1.694 | 1.846 | 1.753 |  |  |
| **Ramachandran plot** |  |  |  |  |  |
| Favoured (%) | 96.15 | 96.05 | 96.34 |  |  |
| Allowed  (%) | 3.85 | 3.95 | 3.56 |  |  |
| Outliers (%) | 0 | 0 | 0.1 |  |  |
| **Validation** |  |  |  |  |  |
| Rotamer outliers  (%) | 0.45 | 0.45 | 0.78 |  |  |
| Molprobity score | 0.96 | 1.10 | 0.97 |  |  |
| Clash score | 0.58 | 1.17 | 0.71 |  |  |

**Supplementary file 1a. Statistics of data collection, 3D reconstruction and validation.**

| **Residue** | **Residue name** | **Mapped name*** | **Total charge** |
| --- | --- | --- | --- |
| 1201 | NAG | YB4 | 0.000 |
| 1202 | NAG | YBV | -0.194 |
| 1203 | MAN | MB0 | 0.194 |
| 1205 | SMA | SG0 | -0.806 |
| 1301 | NAG | YB4 | 0.000 |
| 1302 | NAG | YB0 | 0.194 |
| 1401 | NAG | YB4 | 0.000 |
| 1402 | NAG | YBQ | -0.388 |
| 1403 | MAN | MB0 | 0.194 |
| 1404 | MAN | MB0 | 0.194 |
| 1405 | SMA | SG4 | -1.000 |
| 1406 | GLC | GB0 | 0.194 |
| 1501 | NAG | YB4 | 0.000 |
| 1502 | NAG | YBQ | -0.388 |
| 1503 | MAN | MB0 | 0.194 |
| 1504 | MAN | MB0 | 0.194 |
| 1505 | SMA | SG0 | -0.806 |
| 1601 | NAG | YB4 | 0.000 |
| 1602 | NAG | YBV | -0.194 |
| 1603 | MAN | MB0 | 0.194 |
| 1605 | SMA | SG0 | -0.806 |
| 1701 | NAG | YB4 | 0.000 |
| 1702 | NAG | YBQ | -0.388 |
| 1703 | MAN | MB0 | 0.194 |
| 1704 | MAN | MB0 | 0.194 |
| 1705 | SMA | SG0 | -0.806 |
| 1801 | NAG | YB4 | 0.000 |
| 1802 | NAG | YBQ | -0.388 |
| 1803 | MAN | MB0 | 0.194 |
| 1804 | MAN | MB0 | 0.194 |
| 1805 | SMA | SG4 | -1.000 |
| 1806 | GLC | GB0 | 0.194 |
| 1901 | NAG | YB4 | 0.000 |
| 1902 | NAG | YBQ | -0.388 |
| 1903 | MAN | MB0 | 0.194 |
| 1904 | MAN | MB0 | 0.194 |
| 1905 | SMA | SG0 | -0.806 |
| 2001 | NAG | YB4 | 0.000 |
| 2002 | NAG | YBQ | -0.388 |
| 2003 | MAN | MB0 | 0.194 |
| 2004 | MAN | MB0 | 0.194 |
| 2005 | SMA | SG0 | -0.806 |
| 2101 | NAG | YB4 | 0.000 |
| 2102 | NAG | YBQ | -0.388 |
| 2103 | MAN | MB0 | 0.194 |
| 2104 | MAN | MB0 | 0.194 |
| 2105 | SMA | SG0 | -0.806 |
| 2201 | NAG | YB4 | 0.000 |
| 2202 | NAG | YB0 | 0.194 |
| 2301 | NAG | YB4 | 0.000 |
| 2302 | NAG | YB6 | 0.000 |
| 2303 | MAN | MB0 | 0.194 |
| 2401 | NAG | YB4 | 0.000 |
| 2402 | NAG | YBQ | -0.388 |
| 2403 | MAN | MB0 | 0.194 |
| 2404 | MAN | MB0 | 0.194 |
| 2405 | SMA | SG4 | -1.000 |
| 2406 | GLC | GB0 | 0.194 |
| 2501 | NAG | YB4 | 0.000 |
| 2502 | NAG | YBQ | -0.388 |
| 2503 | MAN | MB0 | 0.194 |
| 2504 | MAN | MB0 | 0.194 |
| 2505 | SMA | SG0 | -0.806 |
| 2601 | NAG | YB4 | 0.000 |
| 2602 | NAG | YBV | -0.194 |
| 2603 | MAN | MB0 | 0.194 |
| 2605 | SMA | SG0 | -0.806 |
| 2701 | NAG | YB4 | 0.000 |
| 2702 | NAG | YBQ | -0.388 |
| 2703 | MAN | MB0 | 0.194 |
| 2704 | MAN | MB0 | 0.194 |
| 2705 | SMA | SG0 | -0.806 |
| 2801 | NAG | YB0 | 0.194 |
| 2901 | NAG | YB4 | 0.000 |
| 2902 | NAG | YBQ | -0.388 |
| 2903 | MAN | MB0 | 0.194 |
| 2904 | MAN | MB0 | 0.194 |
| 2905 | SMA | SG0 | -0.806 |
| 3001 | NAG | YB4 | 0.000 |
| 3002 | NAG | YBQ | -0.388 |
| 3003 | MAN | MB0 | 0.194 |
| 3004 | MAN | MB0 | 0.194 |
| 3005 | SMA | SG0 | -0.806 |

**Supplementary file 1b:** Mapping of glycan residues from the structure file to residues of the GLYCAM force field or the newly charge-derived SG0 and SG4 residues, representing the 1-substituted and 1,4-substituted SMA.

| **Atom** | **Charge** |
| --- | --- |
| C6 | -0.0742 |
| S1 | 1.2319 |
| O6 | -0.7039 |
| O7 | -0.7039 |
| O8 | -0.7039 |
| C5 | 0.1334 |
| O5 | -0.2985 |
| C1 | 0.3664 |
| C2 | 0.0949 |
| O2 | -0.2312 |
| C3 | 0.324 |
| O3 | -0.2189 |
| C4 | 0.1802 |
| O4 | -0.2023 |

**Supplementary file 1c:** RESP charges derived for residue SG0 on the HF/6-31G*//HF/6-31G* level of theory (see Methods for details).

| **Atom** | **Charge** |
| --- | --- |
| C6 | -0.0559 |
| S1 | 1.1863 |
| O6 | -0.6936 |
| O7 | -0.6936 |
| O8 | -0.6936 |
| C5 | 0.1413 |
| O5 | -0.2759 |
| C8 | 0.3583 |
| C2 | 0.1387 |
| O2 | -0.2298 |
| C3 | 0.2118 |
| O3 | -0.1919 |
| C4 | 0.1517 |
| O4 | -0.3538 |

**Supplementary file 1d:** RESP charges derived for residue SG4 on the HF/6-31G*//HF/6-31G* level of theory (see Methods for details).
